# Mindfulness and academic procrastination among Chinese adolescents: a moderated mediation model

**DOI:** 10.3389/fpsyg.2024.1409472

**Published:** 2024-09-02

**Authors:** Pengfei Yue, Jiaxin Zhang, Yumei Jing

**Affiliations:** ^1^College of Education Science, Hubei Normal University, Huangshi, China; ^2^Faculty of Education and Arts, College of Arts and Science of Hubei Normal University, Huangshi, China

**Keywords:** mindfulness, learning vigor, academic procrastination, harsh parenting, trait activation theory

## Abstract

**Background:**

While previous studies have linked mindfulness to reduced academic procrastination, the mechanisms involved remain under-explored. This study deepens the understanding by investigating how learning vigor mediates the mindfulness and procrastination relationship, and how harsh parenting influences this mediation.

**Methods:**

This study, adopting a positivist research approach, utilized a cross-sectional design. Data were collected from 800 students at three middle schools in Henan Province, China, through cluster random sampling. This approach yielded 800 questionnaires. The participants sequentially completed four questionnaires: the Mindfulness Attention Awareness Scale, the Utrecht Work Engagement Scale-Student, the Aitken Procrastination Inventory, and the Harsh Parenting Questionnaire. After removing 67 invalid questionnaires due to incomplete responses and patterned answers, a total of 733 valid questionnaires were obtained, with 53.3% girls and an average age of 13.12 years (SD = 1.01), leading to an effectiveness rate of 91.63%. Upon data collection, SPSS 26.0 software was used for correlation analysis, mediation analysis, and moderated mediation analysis to assess the relationships between variables.

**Results:**

(1) Mindfulness negatively predicts academic procrastination; (2) Learning vigor serves as a mediator in the relationship between mindfulness and academic procrastination; and (3) Harsh parenting moderates the relationship between mindfulness and learning vigor. Specifically, the positive impact of mindfulness on learning vigor is more pronounced in individuals experiencing lower levels of harsh parenting compared to those with higher levels.

**Conclusion:**

This study reveals that mindfulness significantly protects against academic procrastination in adolescents, with 52.27% of this effect mediated by increased learning vigor. Additionally, it shows that high levels of harsh parenting weaken mindfulness’s positive impact on learning vigor, tempering its overall protective influence on procrastination. These insights, which apply Trait Activation Theory to educational psychology, not only deepen our understanding of the dynamics between mindfulness and procrastination but also have important implications for addressing academic procrastination in Chinese adolescents.

## Introduction

In Chinese society, academic achievement is highly valued, and excellent performance brings long-term benefits to individuals and families. However, a concerning trend has emerged: academic procrastination is widespread among students. Academic procrastination refers to the voluntary but irrational postponement of tasks beyond the planned timeframe. According to Steel’s (2007) research, this behavior negatively impacts students’ academic performance. Specifically, academic procrastination can degrade students’ academic outcomes, as demonstrated in studies by Sokic et al. (2022) and Tian et al. (2021).

Moreover, academic procrastination leads to a series of undesirable consequences, including declining grades, negative emotions, cognitive biases (Flett et al., 2016), and problematic mobile phone use (Hong et al., 2021). These effects significantly impact adolescents’ physical and mental development. So, why do students intentionally delay crucial academic tasks? Many scholars have investigated the factors influencing academic procrastination to answer this question.

Research findings indicate that external factors such as stress (Yue and Zhang, 2022; Yue et al., 2023), parenting styles (Li et al., 2023), and peer attachment (Jin et al., 2019), as well as internal factors including learning styles (Wan Hussin and Mohd Matore, 2023), fear of failure (Ahmadi, 2023), impulsivity (Hamvai et al., 2023), perfectionism (Huang et al., 2023), metacognitive beliefs (Safari and Yousefpoor, 2022), and personality types (Ocansey et al., 2022), all contribute to academic procrastination.

With the rise of positive psychology, researchers have begun exploring the relationship between mindfulness and academic procrastination. Studies have found a negative correlation between mindfulness and academic procrastination (Chen, 2019; Serrano et al., 2022). Furthermore, mindfulness interventions have shown significant effects on addressing academic procrastination (Chen, 2012; Su, 2016; Rad Hassan et al., 2023). However, previous research has paid less attention to the internal mechanisms, such as mediation and moderation, in the relationship between mindfulness and academic procrastination. Understanding these internal mechanisms is crucial for a deeper comprehension of the relationship between these variables.

Specifically, exploring mediation mechanisms helps identify key factors that bridge the gap between two variables, while studying moderation mechanisms reveals when the relationship between variables is stronger or weaker. Therefore, this study aims to construct a moderated mediation model to investigate the underlying mechanisms between mindfulness and academic procrastination, enriching our understanding of this relationship.

### Mindfulness and academic procrastination

The Short-term Mood Regulation Theory (STMR) of procrastination proposes that when individuals encounter negative emotions while pursuing goals such as learning tasks, they may forgo long-term beneficial behaviors, including studying, as a means to alleviate their current negative emotions (Sirois and Pychyl, 2013). This behavior often leads to academic procrastination. Mindfulness, on the other hand, is recognized as an effective strategy for emotion regulation (Gawande et al., 2023). It involves a focus on the present moment and a non-judgmental approach to various experiences (Kabat-Zinn, 2003). The Reperceiving Model of Mindfulness suggests that individuals with high mindfulness levels observe the contents of their consciousness more objectively and maintain a transient attitude toward psychological phenomena. Consequently, they demonstrate greater tolerance for unpleasant internal states, such as boredom during learning (Shapiro et al., 2006), thereby reducing the urge to engage in avoidance behaviors like academic procrastination to repair negative emotions. This theory is supported by empirical studies, which show that mindfulness interventions significantly decrease students’ levels of academic procrastination (Chen, 2012; Su, 2016; Rad Hassan et al., 2023). Given the role of mindfulness in emotion regulation and its potential to mitigate academic procrastination, this study proposes Hypothesis 1: Mindfulness negatively predicts academic procrastination.

### The mediating effect of learning vigor

Firstly, mindfulness is positively associated with learning vigor. Learning vigor, characterized by high levels of strength, mental resilience, a willingness to invest effort, and persistence in learning despite difficulties (Schaufeli et al., 2002; Jia et al., 2020), is composed of being alert, alive, and energetic (Song et al., 2015). Highly mindful individuals tend to be more alert and engaged in experiencing the present moment. Additionally, research by Smith et al. (2008) has found that individuals with high mindfulness exhibit higher daily energy levels. Therefore, it stands to reason that individuals with high mindfulness typically demonstrate greater vigor, particularly in learning environments where they are more attentive and dynamic.

Given the positive association between mindfulness and learning vigor discussed above, it is logical to anticipate that learning vigor may also have an impact on academic procrastination. Specifically, learning vigor negatively predicts academic procrastination. Individuals endowed with high learning vigor, who possess greater strength and resilience, are better equipped to exercise self-control in their learning, according to The Strength Model of Self-control. This model posits that self-control consumes internal resources, and its success relies on the available strength in the individual’s reserve (Baumeister et al., 1998). Consequently, learners blessed with elevated vigor are more adept at initiating learning activities, thus mitigating procrastination. Furthermore, individuals with high vigor exhibit stronger psychological resilience, rendering them less inclined to avoid learning challenges through procrastination (Li, 2019; Zhang, 2020).

Based on the aforementioned discussion, this study proposes Hypothesis 2: Learning vigor mediates the negative relationship between mindfulness and academic procrastination. That is, higher mindfulness fosters increased learning vigor, which subsequently leads to a reduction in academic procrastination.

### The moderating effect of harsh parenting

Harsh parenting, typically characterized by disciplinary actions such as spanking, slapping, yelling, and shouting (Hinnant et al., 2015; Wang et al., 2018), significantly impacts children’s learning vigor. According to the Energy and Vitality Model, activities that impede basic psychological needs lead to a decrease in vigor (Ryan and Deci, 2008). Consequently, when parents enforce or control learning activities while neglecting children’s psychological needs, it diminishes their enthusiasm for learning. Empirical studies further support this notion, indicating a negative correlation between harsh parenting and the fulfillment of basic psychological needs (Tang, 2020), thus suggesting that such parenting practices hinder the development of learning vigor.

The Trait Activation Theory (TAT) proposes that harsh parenting, as a constraining situational factor, can suppress the expression of mindfulness traits (Tett and Burnett, 2003). In essence, higher levels of harsh parenting are less conducive to the exercise of mindfulness and weaken its potential positive effect on learning vigor. However, the Conservation of Resources Theory (COR) offers a contrasting perspective (Hobfoll et al., 2018). COR posits that in situations of resource loss, such as the depletion of learning vigor due to harsh parenting, individuals strive to utilize existing resources to acquire new ones, aiming to recuperate from the loss. Therefore, it is plausible that in the face of intense harsh parenting, individuals may actively employ mindfulness to bolster learning vigor, thereby strengthening the influence of mindfulness on learning vigor.

**Figure 1:**
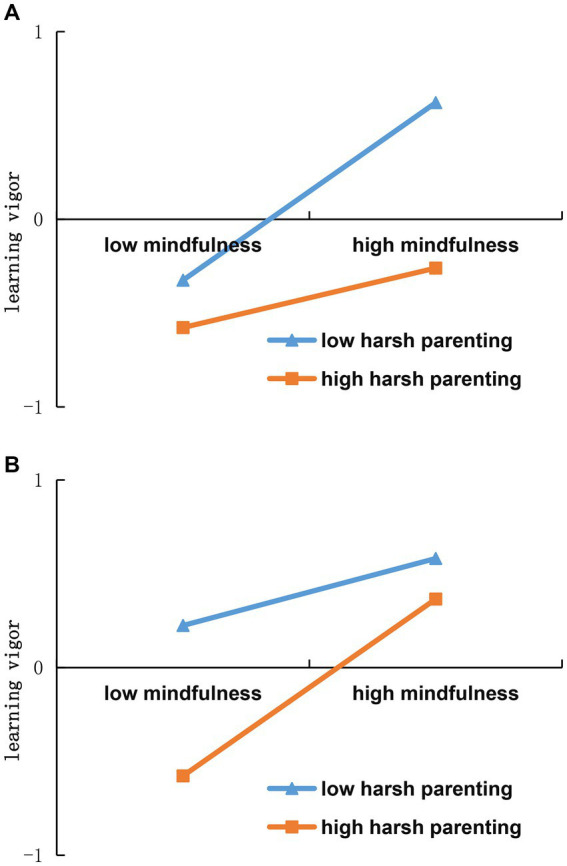
Schematic diagram of the two moderation modes. **(A)** The mode of “trait activation”. **(B)** The mode of “conservation of resources”.

Considering these contrasting theoretical viewpoints, this study proposes Hypothesis 3: Harsh parenting moderates the relationship between mindfulness and learning vigor. Given the scarcity of research exploring the interplay between harsh parenting and mindfulness, this investigation aims to delve into the moderation mechanisms encompassing both the “trait activation” and “conservation of resources” frameworks. This exploration will be conducted through an exploratory analysis, without preconceived hypotheses. For a visual representation of these two moderating mechanisms, refer to Figures 1A,B, which illustrate schematic diagrams of each mode.

### The current study

**Figure 2:**
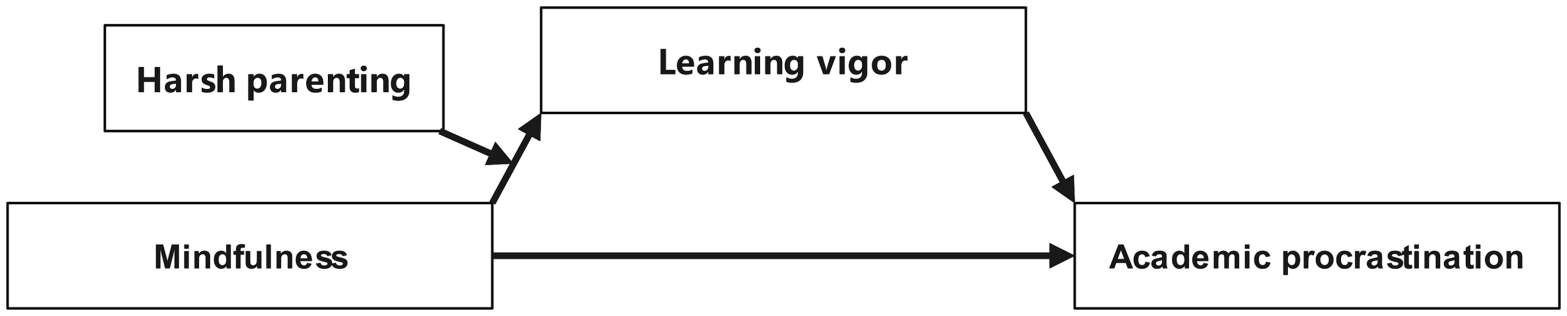
Hypothetical moderated mediation model.

This study presents a hypothetical moderated mediation model (illustrated in Figure 2) to achieve three key goals: first, to assess the correlation between mindfulness and academic procrastination; second, to investigate learning vigor’s mediating role in this correlation; and third, to scrutinize how environmental factors like harsh parenting and individual traits such as mindfulness jointly affect learning vigor, thereby influencing adolescent academic procrastination. The model strives to deepen our comprehension of mindfulness’s impact on procrastination, elucidating its mechanisms and the conditions that intensify or diminish its effects. Ultimately, this study aims to offer valuable insights for preventing and addressing academic procrastination among Chinese adolescents.

## Materials and methods

### Research design

The research approach of this study is based on positivism. A cross-sectional design was used to conduct a questionnaire survey on participants. They sequentially completed four questionnaires: the Mindfulness Attention Awareness Scale, the Utrecht Work Engagement Scale-Student, the Aitken Procrastination Inventory, and the Harsh Parenting Questionnaire. After data collection, correlation analysis, mediation analysis, and moderated mediation analysis were employed to examine the relationships between variables.

### Participants

In this study, data were collected at a single time point from 800 students at three middle schools in Henan Province, China, using cluster random sampling, resulting in 800 questionnaires for the survey. Invalid questionnaires were removed from the recovered questionnaires based on the following criteria: (1) Respondents showing obvious patterns in their answers, such as a repeating sequence of “12,345”; (2) Respondents selecting extreme options for all questions, for instance, choosing the highest or lowest score for every question; and (3) Respondents completing the questionnaires in an unreasonably short period of time. In this study, 2 min was predetermined as a reasonable answering time, and data with less than 2 min of answering time were excluded. Finally, 733 valid questionnaires were obtained, resulting in an effectiveness rate of 91.63%. Among the valid responses, the average age of participants was 13.12 years (SD = 1.01), comprising 342 boys (46.7%) and 391 girls (53.3%). The distribution across grades was 354 students (48.3%) in 7th grade, 177 (24.1%) in 8th grade, and 202 (27.6%) in 9th grade. The sample included 46 only children (6.3%) and 687 non-only children (93.7%), with 617 (84.2%) rural students and 116 (15.8%) urban students. To provide a better picture of the demographic profile, we have presented the details in Table 1.

**Table 1:** Participant demographics.

Variable	Category	Frequency	Percentage
Gender	Male	342	46.7%
	Female	391	53.3%
Grade	7th grade	354	48.3%
	8th grade	177	24.1%
	9th grade	202	27.6%
Only child status	Only child	46	6.3%
	Non-only child	687	93.7%
Family location	Rural	617	84.2%
	Urban	116	15.8%

### Research tools

#### Mindfulness Attention Awareness Scale

The Mindfulness Attention Awareness Scale (MAAS), originally developed by Brown and Ryan (2003) and later revised by Chen et al. (2012), is a tool used in this study to measure mindfulness. This questionnaire comprises 15 items, each assessing the respondent’s focus on present experiences, exemplified by statements like “I cannot concentrate very well on what is happening in the present.” The scale operates on a single-dimensional structure and uses a 6-point scoring system, where a higher score indicates a higher level of mindfulness. The MAAS has demonstrated good psychometric properties in previous mindfulness research (Huang and Zhao, 2020; Zhang and Yue, 2021). In the current study, the Cronbach’s *α* coefficient for the Mindfulness Attention Awareness Scale was 0.88, indicating high reliability.

#### Utrecht Work Engagement Scale-Student

The Utrecht Work Engagement Scale-Student (UWES-S), initially developed by Schaufeli et al. (2002) and later revised by Fang et al. (2008), is utilized in this study to measure learning vigor. This questionnaire contains 17 items, such as “I can continue studying for very long periods at a time,” and is divided into three dimensions: vigor, dedication, and absorption. For the purpose of this study, only the vigor dimension was employed to assess the level of learning vigor. The scale uses a 7-point scoring system, where a higher score indicates a higher level of learning vigor. Previous studies on learning engagement have demonstrated the good psychometric properties of this scale (Li, 2018; Yue et al., 2022). In this research, the Cronbach’s *α* coefficient for the vigor dimension of the UWES-S was 0.89, reflecting high reliability.

#### Aitken Procrastination Inventory

The Aitken Procrastination Inventory (API), originally developed by Aitken (1982) and later revised by Liu and Lu (2011), is used in this study to assess academic procrastination. The questionnaire comprises 13 items, such as “I always wait until I cannot put off learning tasks any longer.” It is structured into two dimensions: task aversion and fear of failure. The API utilizes a 5-point scale, where a higher score indicates more severe academic procrastination. This instrument has shown good psychometric properties in previous studies focused on academic procrastination (Yue and Zhang, 2022; Yue et al., 2023), indicating its effectiveness and reliability in measuring this phenomenon. In the current research, the Cronbach’s *α* coefficient for the Aitken Procrastination Inventory was 0.74, signifying an acceptable level of internal consistency.

#### Harsh Parenting Questionnaire

In this study, the measurement of harsh parenting was based on a four-question format, drawing from the works of Wang (2017). These questions probe into the adolescents’ experiences with their parents, such as “When I did something wrong or made my parents angry, he (or she) would lose temper or even yell at me,” asking the respondents to rate their parents’ behaviors. The scale used for this questionnaire is a 5-point system, where a higher score indicates a greater degree of harsh parenting as perceived by the adolescents. The reliability of this Harsh Parenting Questionnaire in the current study is indicated by its Cronbach’s *α* coefficient, which is 0.78. This coefficient suggests a good level of internal consistency for the questionnaire, ensuring that it is a reliable tool for assessing the degree of harsh parenting in the context of this research.

### Procedure

The questionnaire data for this study was collected in October 2020. Prior to administering the questionnaire, moral education teachers at the schools provided comprehensive training to the head teachers of each class regarding the test administration procedures and important precautions. The head teachers informed the parents about the test, and both parents and students voluntarily signed informed consent forms. During the actual test, the head teachers organized the distribution of paper questionnaires to the students, who completed them during class meeting times. Participants were guided to answer honestly and reflectively, based on their real-life situations, with an emphasis on the confidentiality of their personal information. The questionnaires were completed within approximately 20 min. Upon completion of the test, the head teachers collected and securely maintained the confidentiality of the questionnaires. The study adhered to the principles of the Declaration of Helsinki and was approved by the Research Ethics Committee at the College of Education Science, Hubei Normal University.

### Data analysis

The collected data were inputted and managed using SPSS 26.0 software, with which descriptive statistical analysis and correlation analysis were conducted. After standardizing the scores of each scale, the two models under investigation were tested using the PROCESS macro program (Hayes, 2013). Specifically, Model 4 was employed to assess the mediating effect of learning vigor, and Model 7 was utilized to evaluate the moderating effect of harsh parenting.

## Results

### Common method biases test

Given the potential for common method biases due to self-reported data collection, measures like anonymous responses and reverse-scoring items were implemented. Additionally, the Harman single factor test was conducted. An unrotated exploratory factor analysis indicated that the first factor accounted for only 20.38% of the variance, significantly below the 40% threshold (Podsakoff et al., 2003), suggesting minimal common method biases in this study.

### Preliminary analyses

According to Table 2, mindfulness positively correlated with learning vigor (*r* = 0.29, *p* < 0.01) and negatively correlated with academic procrastination (*r* = −0.26, *p* < 0.01) and harsh parenting (*r* = −0.23, *p* < 0.01). Learning vigor showed negative correlations with both academic procrastination (*r* = −0.47, *p* < 0.01) and harsh parenting (*r* = −0.20, *p* < 0.01), whereas academic procrastination exhibited a positive correlation with harsh parenting (*r* = 0.15, *p* < 0.01). Furthermore, age was significantly related to academic procrastination (*r* = 0.09, *p* < 0.05), and grade correlated notably with mindfulness (*r* = −0.09, *p* < 0.05), academic procrastination (*r* = 0.10, *p* < 0.01), and harsh parenting (*r* = −0.08, *p* < 0.05). Being an only child was significantly associated with harsh parenting (*r* = −0.14, *p* < 0.01), and family location had a notable correlation with learning vigor (*r* = −0.09, *p* < 0.05). No significant correlations were found between gender and the study variables. According to Wen (2017), demographic variables that are significantly correlated with study variables should be taken as control variables. Thus, age, grade, only child status, and family location were included as control variables in subsequent analyses. To improve the conciseness of Table 2, the correlation coefficient between the demographic variables and the study variables has been omitted from the presentation.

**Table 2:** Descriptive statistics and correlation analysis results for each variable (*n* = 733).

Variables	M ± SD	1	2	3	4
1. Mindfulness	3.66 ± 0.99	1			
2. Learning vigor	3.69 ± 1.34	0.29**	1		
3. Academic procrastination	2.46 ± 0.62	−0.26**	−0.47**	1	
4. Harsh parenting	1.77 ± 0.78	−0.23**	−0.20**	0.15**	1

**p < 0.01.

### Mindfulness and academic procrastination: moderated mediation effect

Following the methods of Hayes (2013) and Wen and Ye (2014), all variables were standardized, and age, grade, only child status, and family location were used as control variables to analyze the moderated mediation effect. The analysis was conducted using the SPSS 26.0 macro program PROCESS, employing the percentile Bootstrap method with deviation correction for testing. This involved 5,000 repeated samples and the calculation of 95% confidence intervals, with specific results presented in Table 3.

**Table 3:** Mindfulness and academic procrastination: moderated mediation effect.

Predictive variable	Learning vigor			Academic procrastination			Learning vigor		
	*β*	*SE*	*t*	*β*	*SE*	*t*	*β*	*SE*	*t*
Mindfulness	0.30	0.04	8.31**	−0.12	0.03	−3.55**	0.26	0.04	7.14**
Learning vigor				−0.45	0.03	−13.17**			
Harsh parenting							−0.17	0.04	−4.55**
Mindfulness × Harsh parenting							−0.13	0.03	−3.88**
*R* ^2^		0.10			0.25			0.14	
*F*		16.60**			41.30**			16.26**	

**p < 0.01.

The first step involved testing the simple mediation model (model 4). Regression analysis revealed that mindfulness significantly negatively predicted academic procrastination (*β* = −0.25, *p* < 0.01). Including learning vigor in the regression equation, mindfulness continued to negatively predict academic procrastination (*β* = −0.12, *p* < 0.01), positively predicted learning vigor (*β* = 0.30, *p* < 0.01), and learning vigor negatively predicted academic procrastination (*β* = −0.45, *p* < 0.01). The mediation effect *a* × *b* = −0.13, Boot SE = 0.02, with a 95% confidence interval [−0.18, −0.10], excluding 0, signifying a significant mediating role of learning vigor between mindfulness and academic procrastination, accounting for 52.27% of the total effect.

The second step tested the moderated mediation effect (model 7). Regression analysis showed that mindfulness positively predicted learning vigor (*β* = 0.26, *p* < 0.01), and harsh parenting negatively predicted learning vigor (*β* = −0.17, *p* < 0.01). The interaction of mindfulness and harsh parenting significantly predicted learning vigor (*β* = −0.13, *p* < 0.01), with a 95% confidence interval [−0.19, −0.06], excluding 0. These results indicated that harsh parenting moderates the relationship between mindfulness and learning vigor.

**Figure 3:**
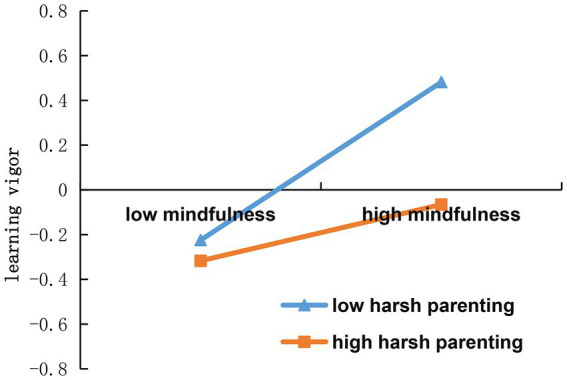
Harsh parenting as a moderator in the relationship between mindfulness and learning vigor.

To explore the moderating role of harsh parenting on the impact of mindfulness on learning vigor, participants were divided into groups based on their harsh parenting levels (±1 SD). A simple slope test was performed, and a simple effect analysis chart was generated (Figure 3). The findings revealed that at low levels of harsh parenting (−1SD), the influence of mindfulness on learning vigor was more pronounced (*b* simple = 0.38, *p* < 0.05), with a 95% confidence interval of [0.29, 0.47]. Conversely, at high levels of harsh parenting (+1SD), this effect was less significant (*b* simple = 0.13, *p* < 0.05), with the 95% confidence interval at [0.03, 0.23].

## Discussion

### Mindfulness and academic procrastination

This study discovered that mindfulness negatively predicts academic procrastination, supporting the predictions of the Short-term Mood Regulation Theory (STMR) of procrastination and aligning with previous research findings (Chen, 2019; Serrano et al., 2022). Additionally, the relationship between mindfulness and academic procrastination can be explained using the Self-regulatory Executive Functioning Model. This model suggests that poor attention control makes task handling more difficult for individuals, such as in learning tasks, leading to a propensity to delay studies (Fernie et al., 2016). However, individuals with high mindfulness possess stronger attention control abilities and exhibit higher learning efficiency (Anderson et al., 2007). For them, learning is easier, and thus, they are less likely to procrastinate.

### The mediating role of learning vigor

This study significantly contributes to the understanding of how mindfulness impacts academic procrastination by highlighting the role of learning vigor. It was found that the effect of mindfulness on academic procrastination is mediated by an improvement in learning vigor, with the mediating effect accounting for 52.27% of the total effect. This substantial percentage indicates that learning vigor explains a considerable portion of the variation between mindfulness and academic procrastination.

The process through which mindfulness affects academic procrastination is twofold. Firstly, mindfulness helps individuals improve their strength levels and maintain a presence in the moment during learning, thereby promoting learning vigor. Secondly, individuals with high learning vigor exhibit energy and strong psychological resilience. They do not shy away from learning difficulties and are less likely to resort to avoidance strategies like academic procrastination. This aligns with findings by Baumeister et al. (1994), who noted that tasks feel more painful and challenging with diminished strength, and Burka and Yuen (1983), who observed increased difficulty in task initiation when individuals are tired.

Previous research has identified different types of self-control abilities (Chen, 2019; Chen, 2012), such as emotional regulation, rumination (cognitive control), and behavioral control, as mediators in the relationship between mindfulness and academic procrastination. Building on The Strength Model of Self-control (Baumeister et al., 1998), this study hypothesized and confirmed that vigor (specifically, learning vigor) is also a crucial mediating mechanism in this relationship. This finding provides a novel explanation for the connection between mindfulness and academic procrastination and enriches the research on the mediating mechanisms through which mindfulness influences academic procrastination.

### The moderating effect of harsh parenting

This study also discovered that harsh parenting moderates the relationship between mindfulness and learning vigor. Specifically, the positive predictive effect of mindfulness on learning vigor was greater in individuals experiencing lower levels of harsh parenting compared to those with higher levels. In other words, the influence of mindfulness on reducing academic procrastination was stronger in individuals with a lower degree of harsh parenting than in those with a higher degree. This finding addresses when the effect of mindfulness on academic procrastination is “stronger” or “weaker” and explores the boundary conditions of this effect. This result aligns with the predictions of Trait Activation Theory, suggesting that harsh parenting inhibits the expression of mindfulness traits, thereby weakening the impact of mindfulness on learning vigor in such environments.

Additionally, Hirst et al. (2011) observed a similar phenomenon in a management study on employee creativity. They found that organizational centralization limited the expression of individual learning orientation, with a lower degree of learning orientation activation in centralized atmospheres. Consequently, the higher the centralization, the weaker the impact of learning orientation on employee creativity, and vice versa. This study applies both TAT and COR to examine the joint effect of harsh parenting and adolescent mindfulness on learning vigor (Hobfoll et al., 2018; Tett and Burnett, 2003). It found that harsh parenting and adolescent mindfulness influence learning vigor through a “trait activation” interaction model, subsequently affecting adolescent academic procrastination. This demonstrates the predictive power of TAT in learning contexts.

### Limitations and future recommendations

Although this study is grounded in solid theory and prior research, it has certain limitations. Firstly, while it identified learning vigor as a partial mediator between mindfulness and academic procrastination and found that the relationship between mindfulness and learning vigor is moderated by harsh parenting, there may still be other mediating and moderating variables between mindfulness and academic procrastination that remain undiscovered. Secondly, the study’s cross-sectional design limits causal inferences. Thirdly, the participants were exclusively from Chinese schools, so the applicability of the findings in other countries and regions requires further validation.

Future research could address these limitations. Firstly, exploring additional, yet undiscovered, mediating and moderating variables between mindfulness and academic procrastination would provide a more comprehensive understanding of their relationship. Secondly, employing a longitudinal design to collect data at multiple time points and conducting cross-lagged analyses could further test the causal relationships between variables. Lastly, researchers in other countries or regions could replicate this study to examine the applicability of its findings in different cultural contexts.

### Research implications

Despite its limitations, this study holds significant importance both theoretically and practically.

Theoretical implications: this study extends beyond the scope of previous research that primarily examined the relationship between mindfulness and academic procrastination from the perspective of self-control ability. By investigating the boundary conditions of mindfulness’s effect on academic procrastination, it enhances the understanding of how mindfulness operates in this domain. Additionally, the study innovatively applies TAT, a concept typically utilized in organizational management, to the field of educational psychology. This application broadens the theoretical framework’s applicability, demonstrating its relevance and utility in a new context.

Practical implications: this research underscores the significance of mindfulness in mitigating academic procrastination among adolescents. Studies indicate that various methods, such as engaging in a short-term virtual-reality based mindfulness breathing meditation (Wagner and Wieczorek, 2024), maintaining a mindfulness journal (Pennebaker and Seagal, 1999), or participating in a Mindfulness-Based Stress Reduction Group (Darehzereshki et al., 2022), can effectively elevate individuals’ levels of mindfulness. The research reveals that boosting learning vigor—which can be strengthened through mindfulness, physical activities, and exposure to nature (Frederick and Ryan, 1995; Tyrväinen et al., 2014)—is pivotal in tackling procrastination. Striking a balance between academic and outdoor activities is crucial, and it is imperative that educational policies facilitate this equilibrium. Additionally, the study identifies that harsh parenting diminishes the efficacy of mindfulness, emphasizing the need for parents to embrace more supportive parenting techniques. In summary, tackling academic procrastination necessitates the cultivation of mindfulness, augmentation of learning vigor, and the adoption of moderate parenting styles.

## Conclusion

This study yielded several key findings:

1. Mindfulness negatively predicts academic procrastination, indicating that higher levels of mindfulness are associated with lower levels of academic procrastination.
2. Learning vigor serves as a mediator in the relationship between mindfulness and academic procrastination. This suggests that mindfulness enhances learning vigor, which in turn reduces the tendency toward academic procrastination.
3. Harsh parenting moderates the relationship between mindfulness and learning vigor. Specifically, the positive impact of mindfulness on learning vigor is more pronounced in individuals experiencing lower levels of harsh parenting compared to those with higher levels. This implies that the effectiveness of mindfulness in reducing academic procrastination is stronger in environments with lower levels of harsh parenting than in those with higher levels of such parenting.

## Data Availability

The raw data supporting the conclusions of this article will be made available by the authors, without undue reservation.
